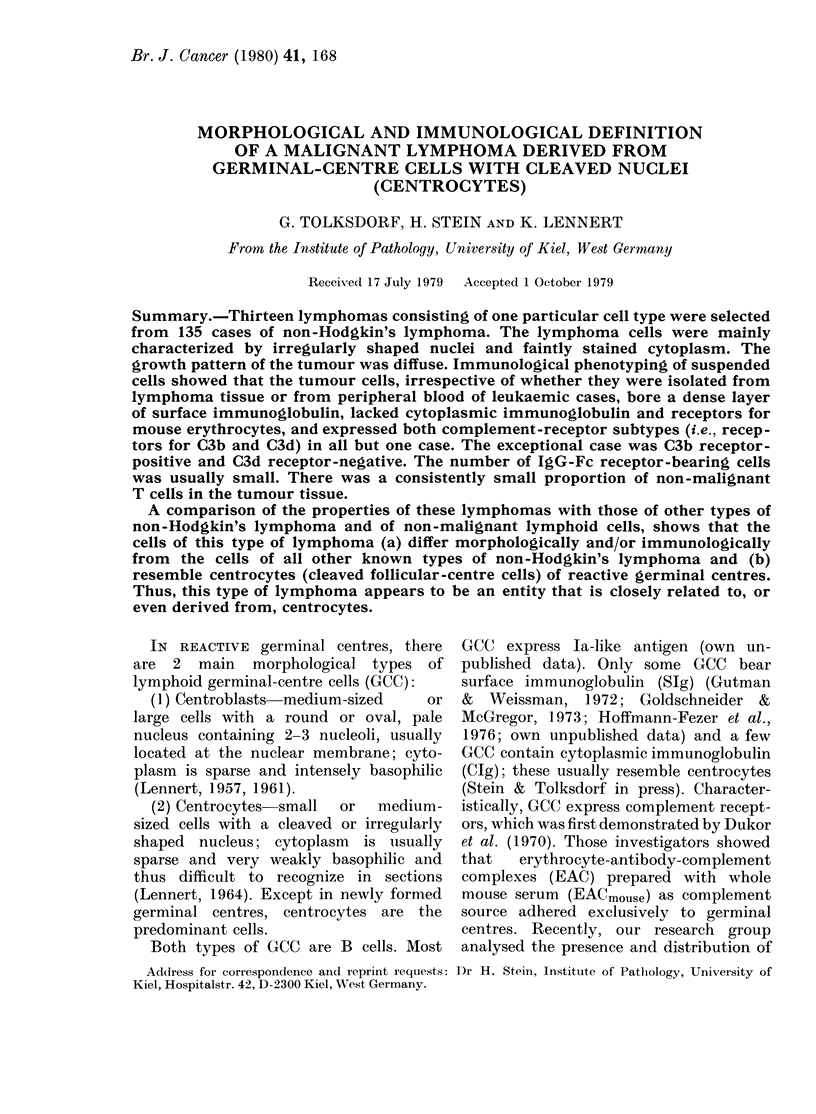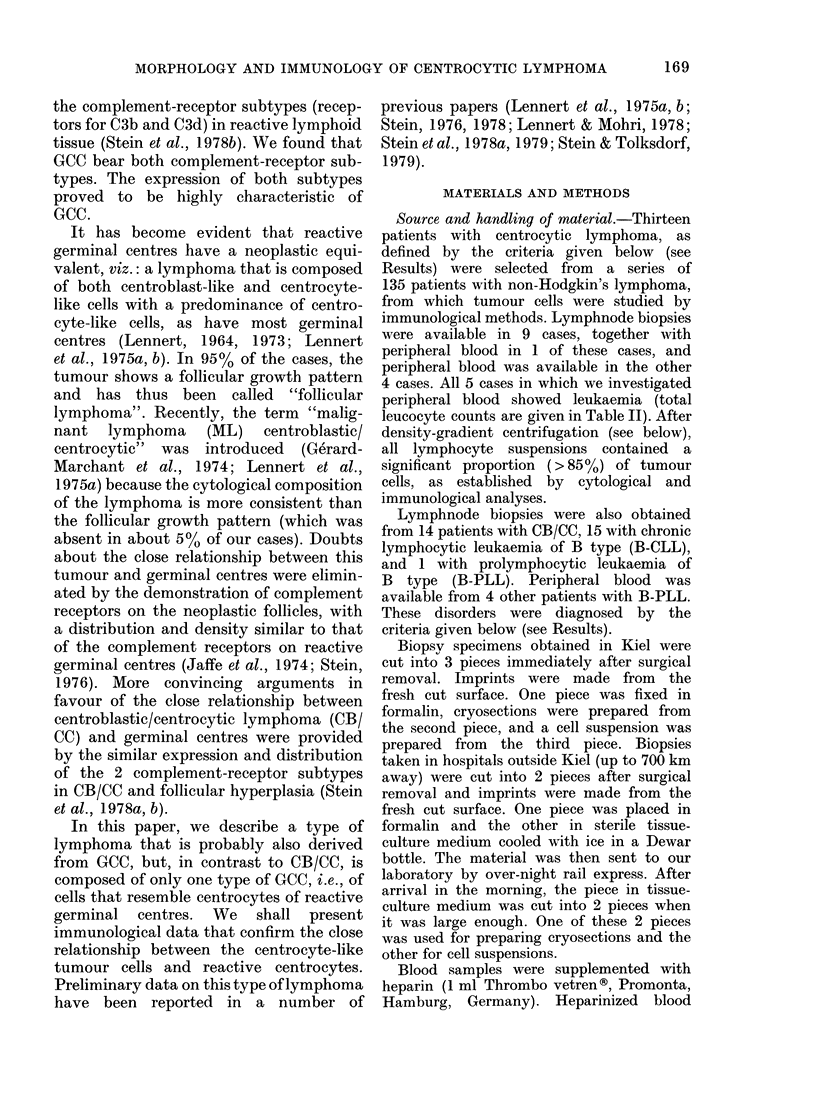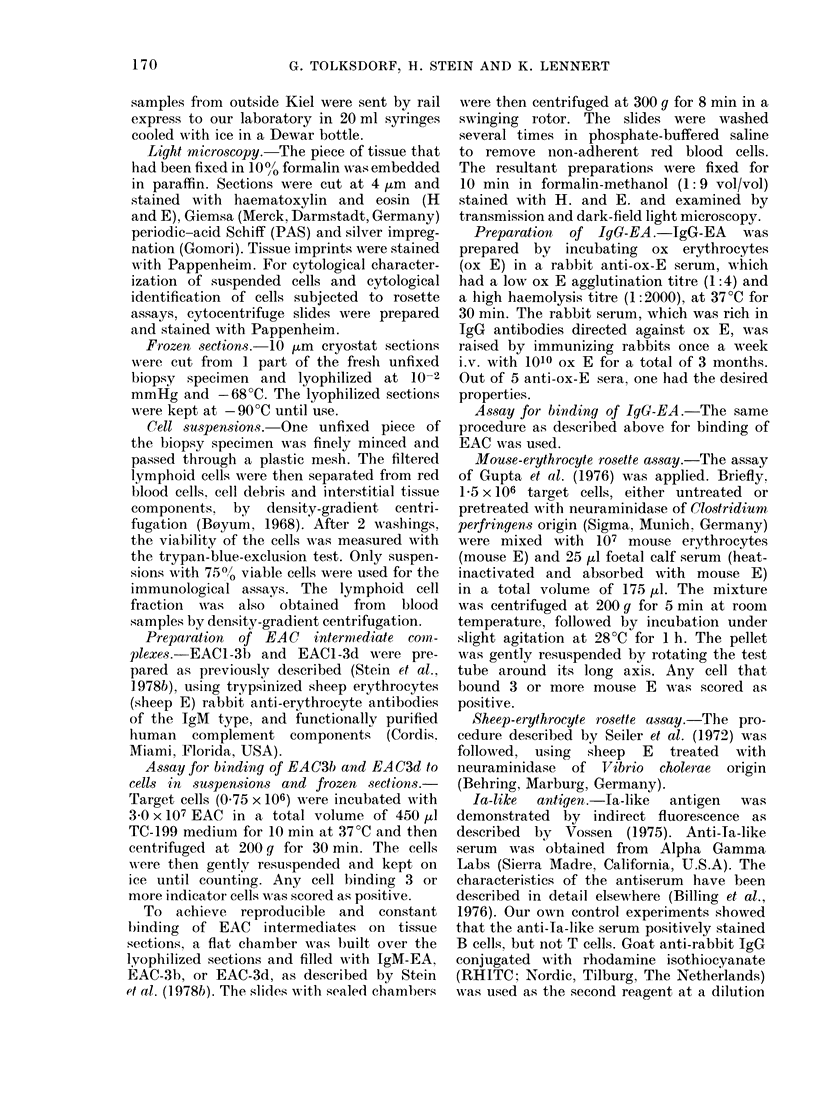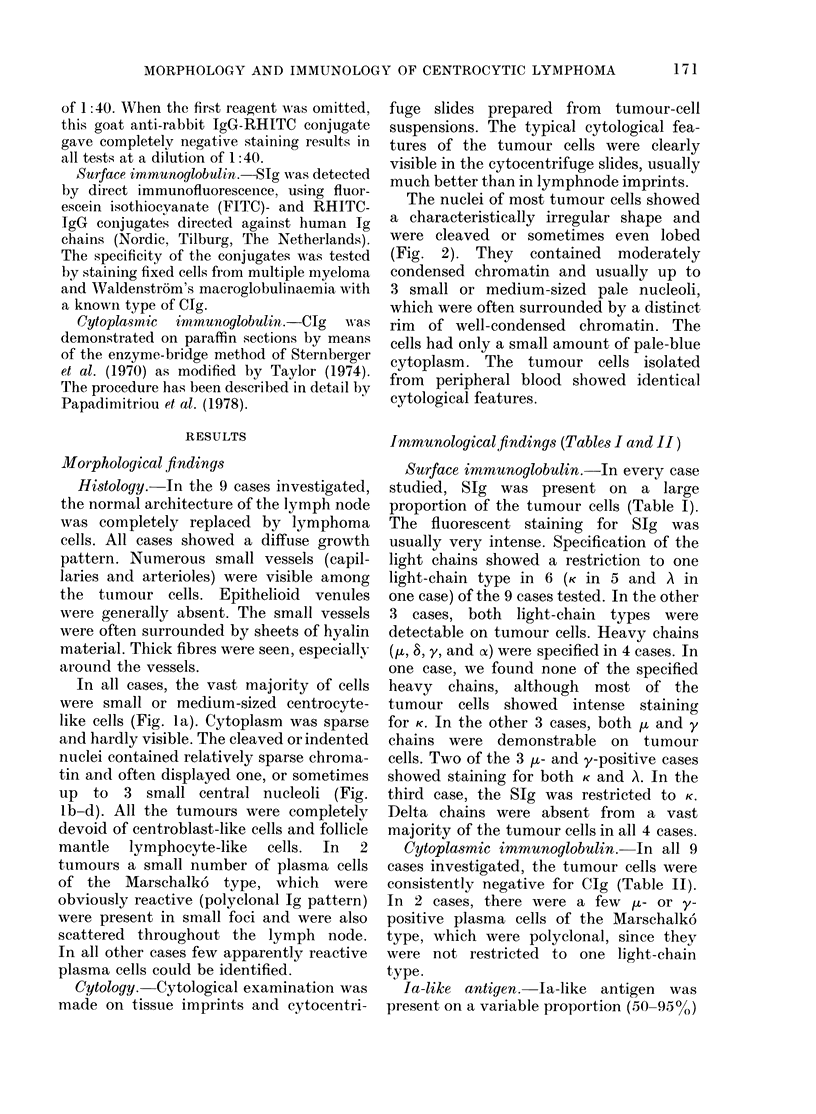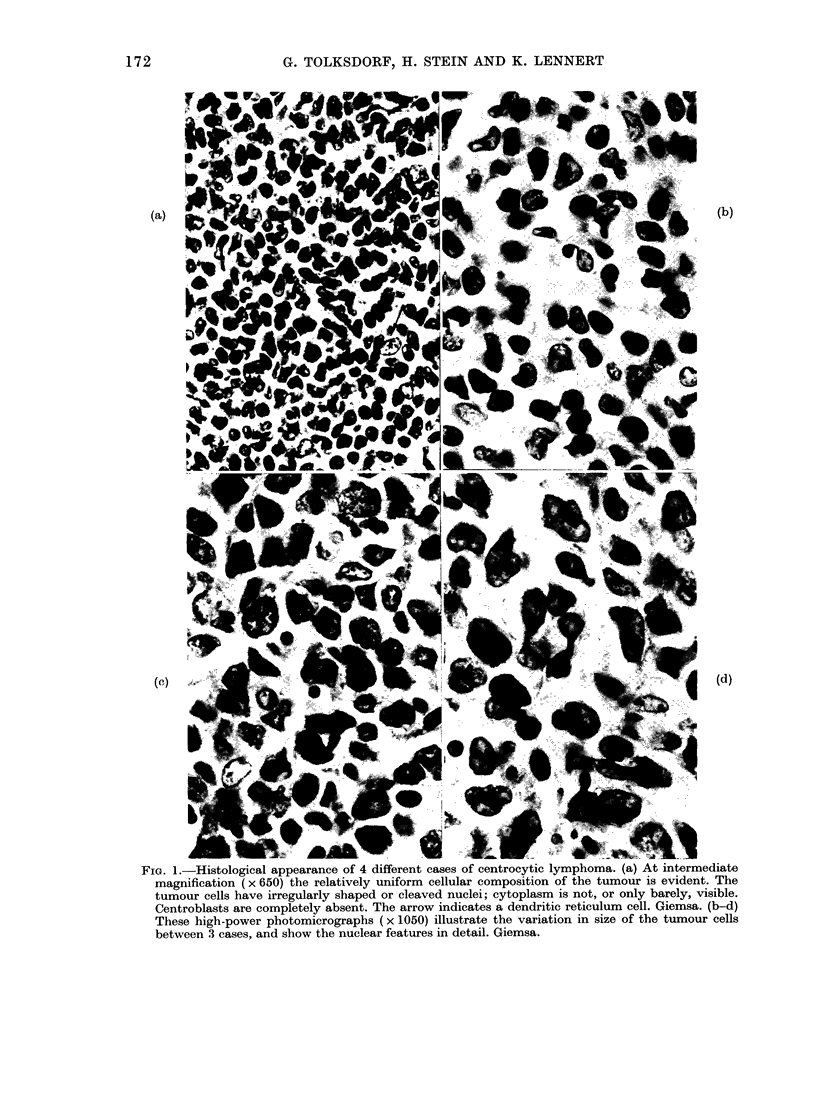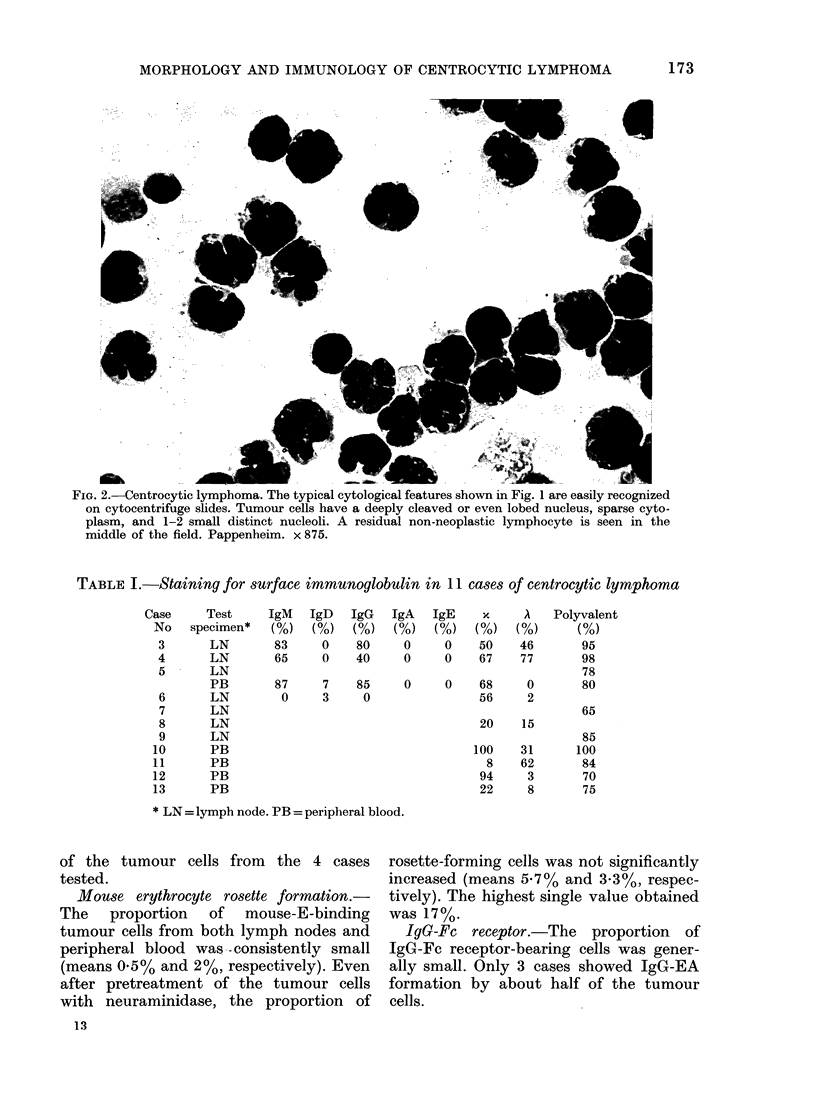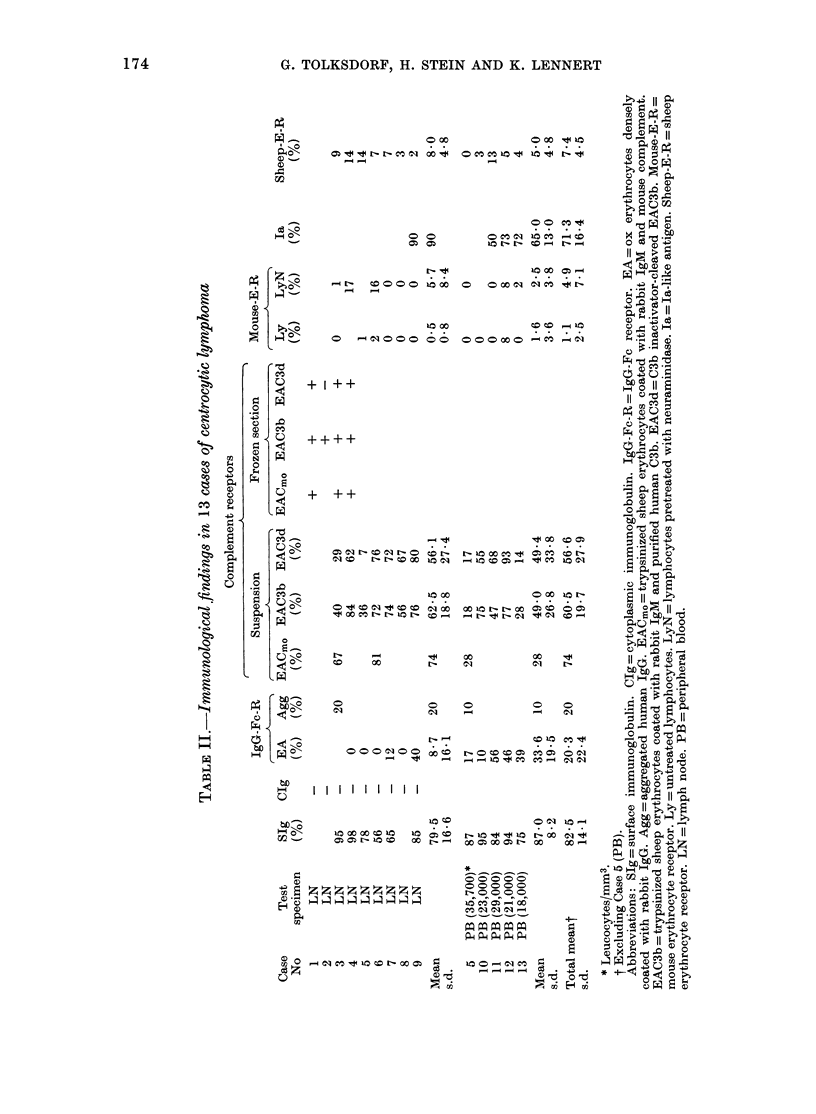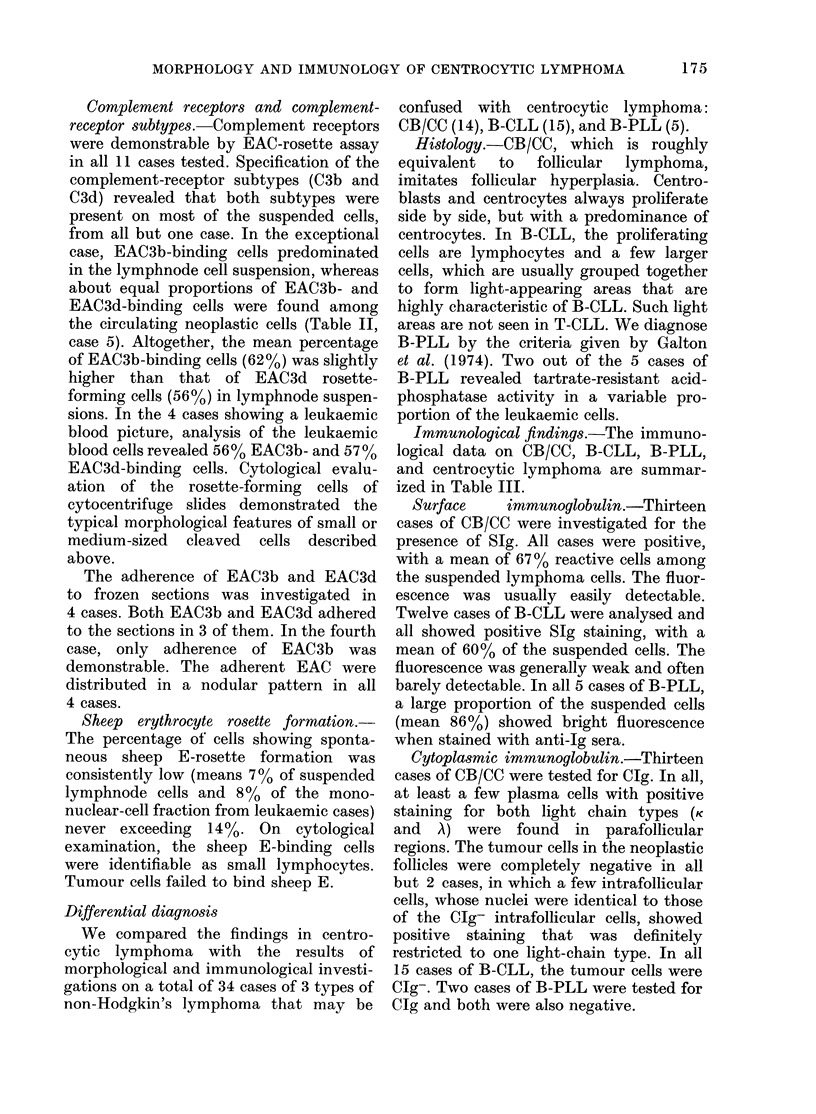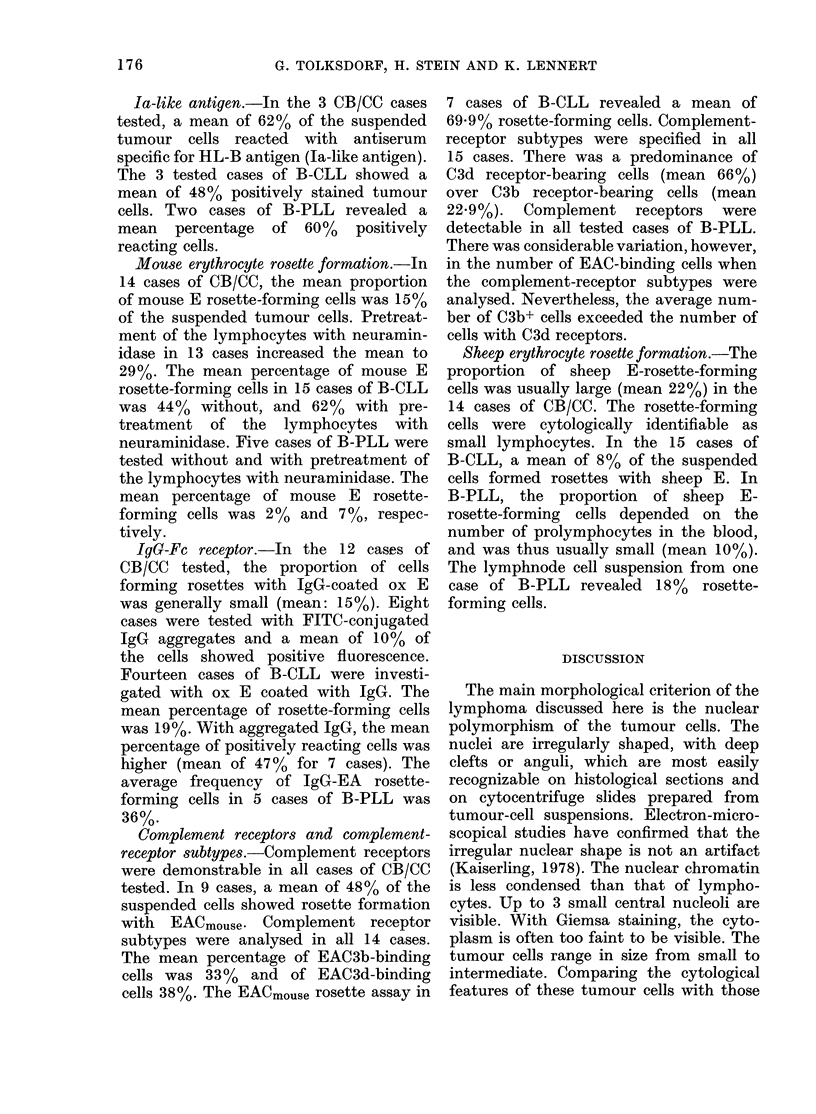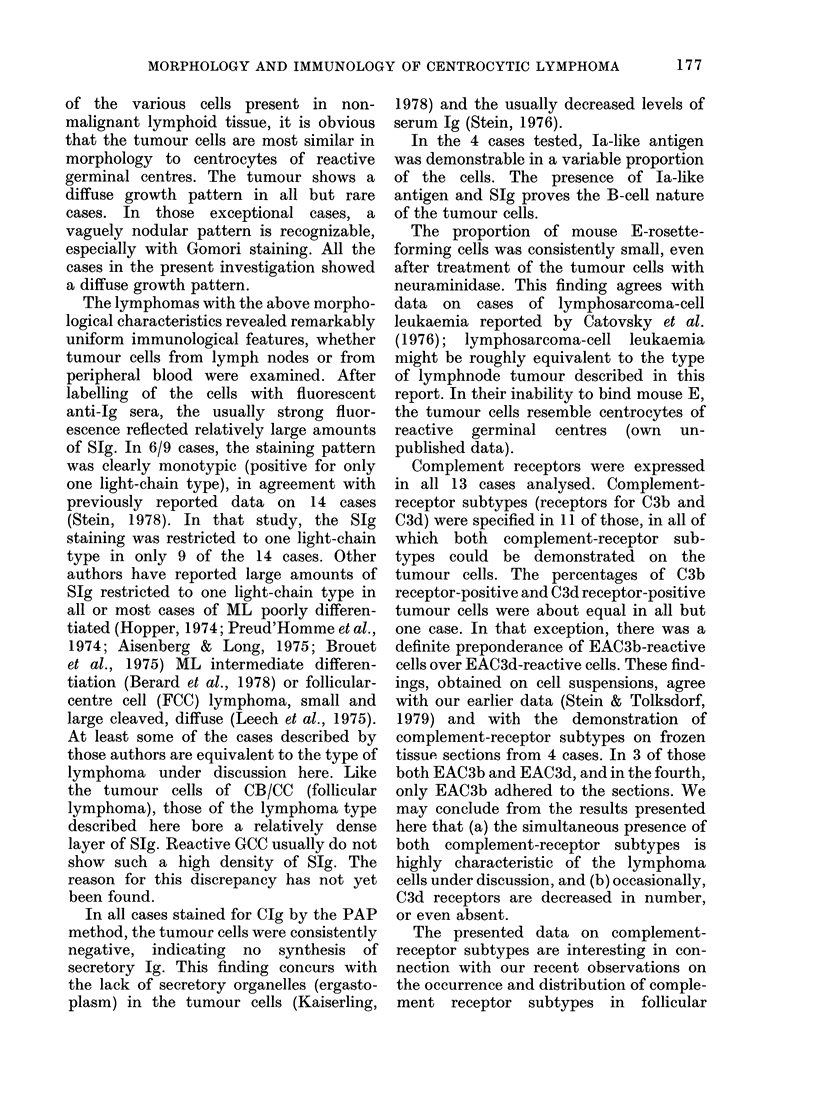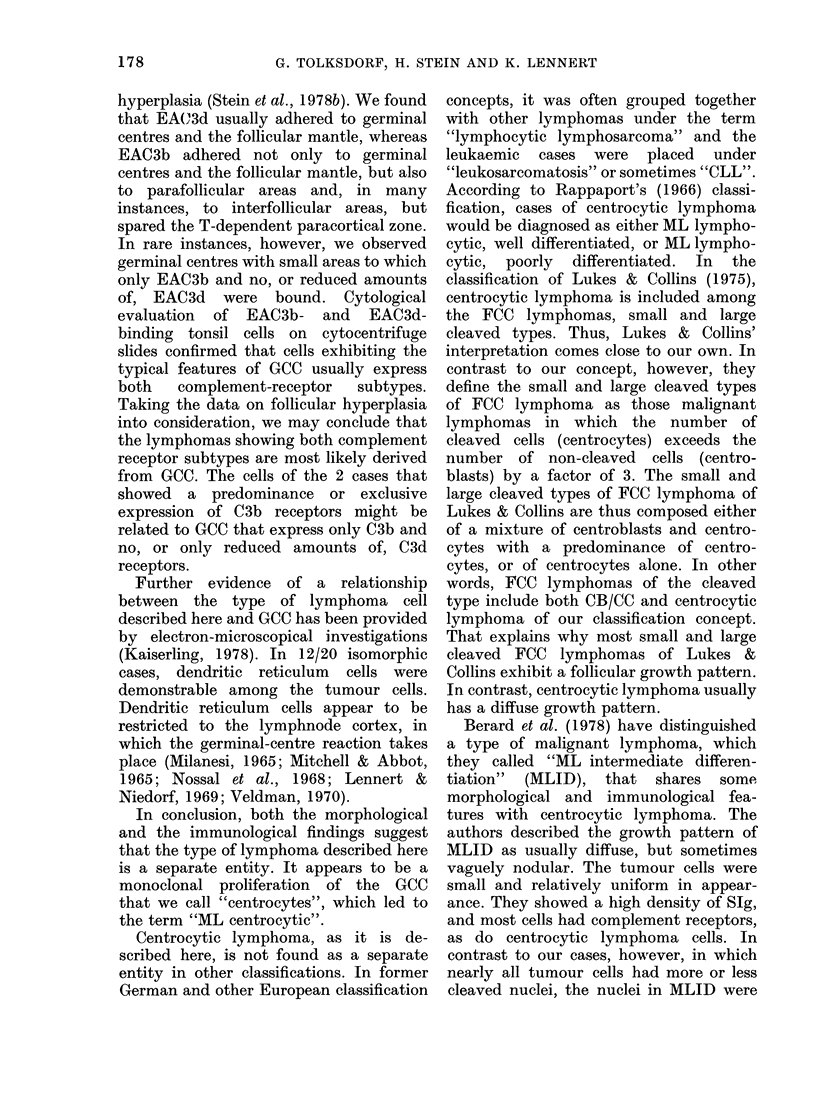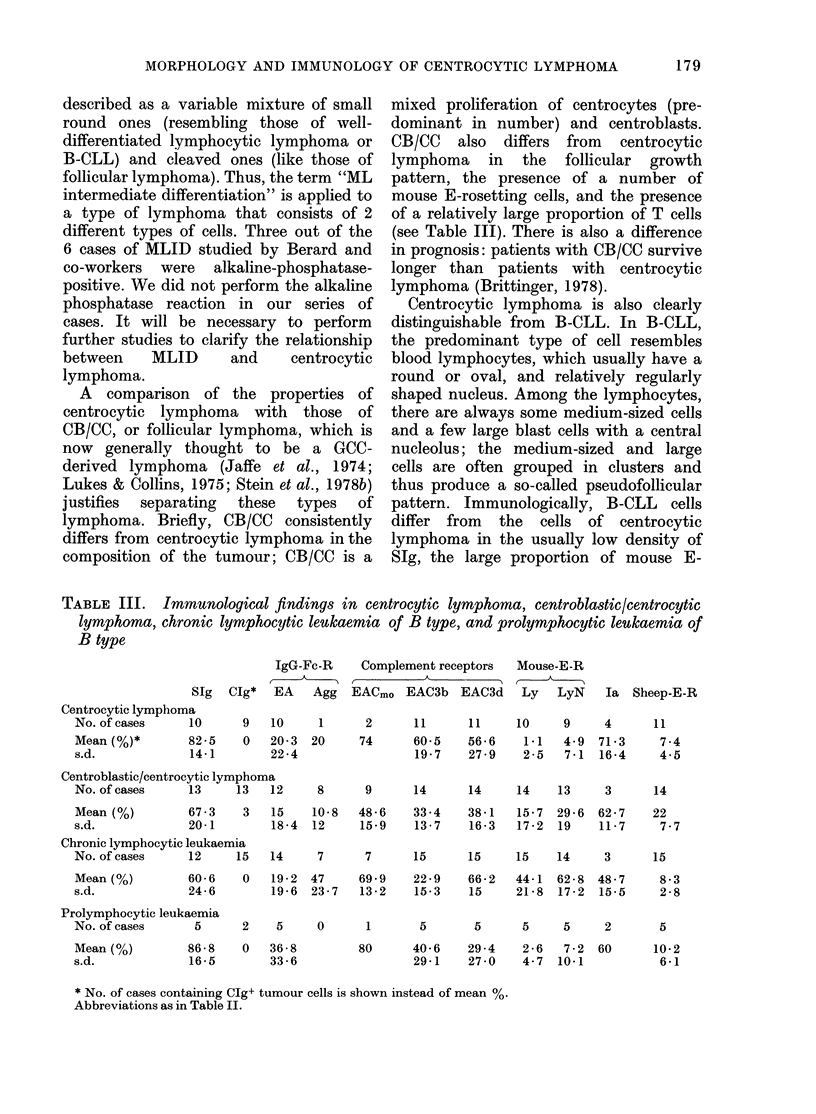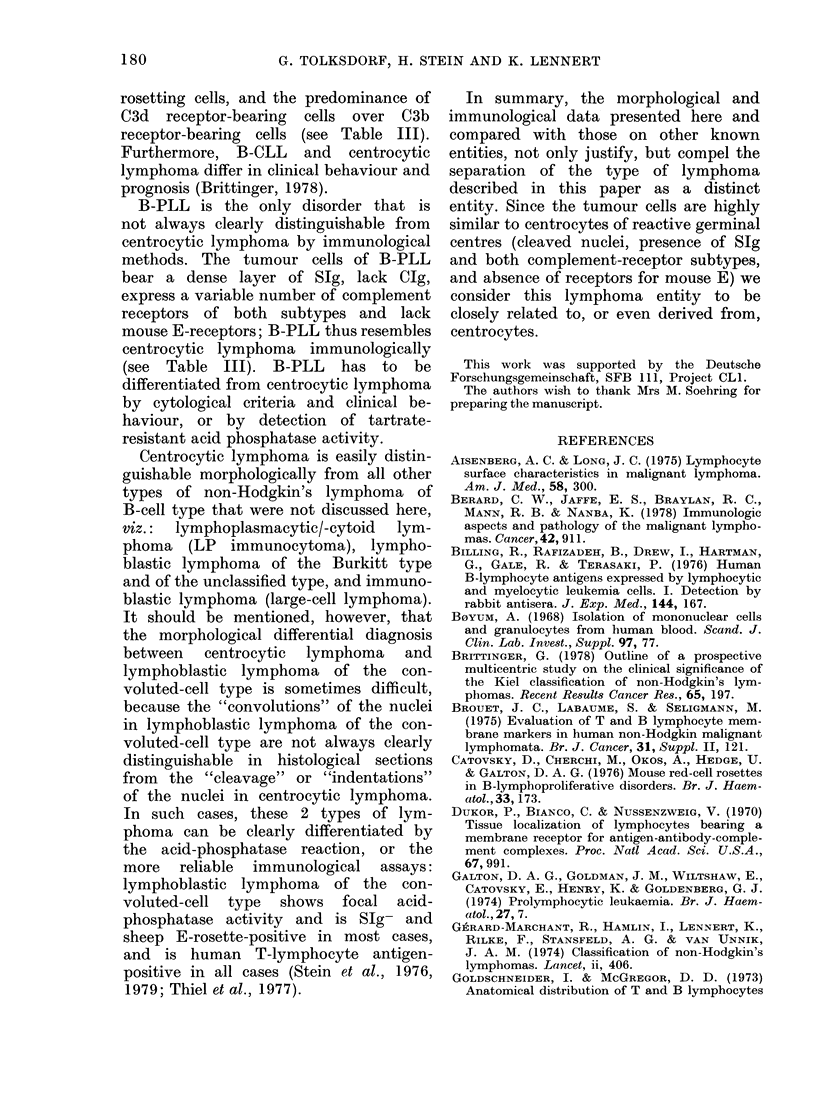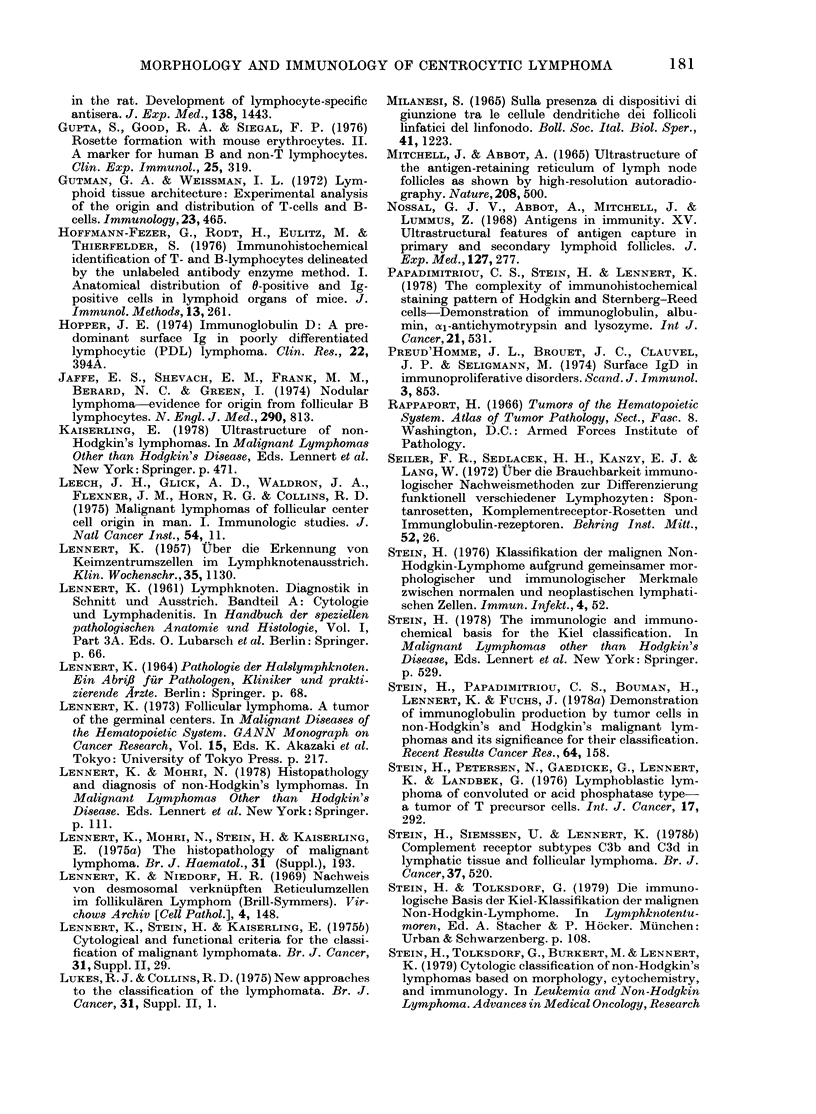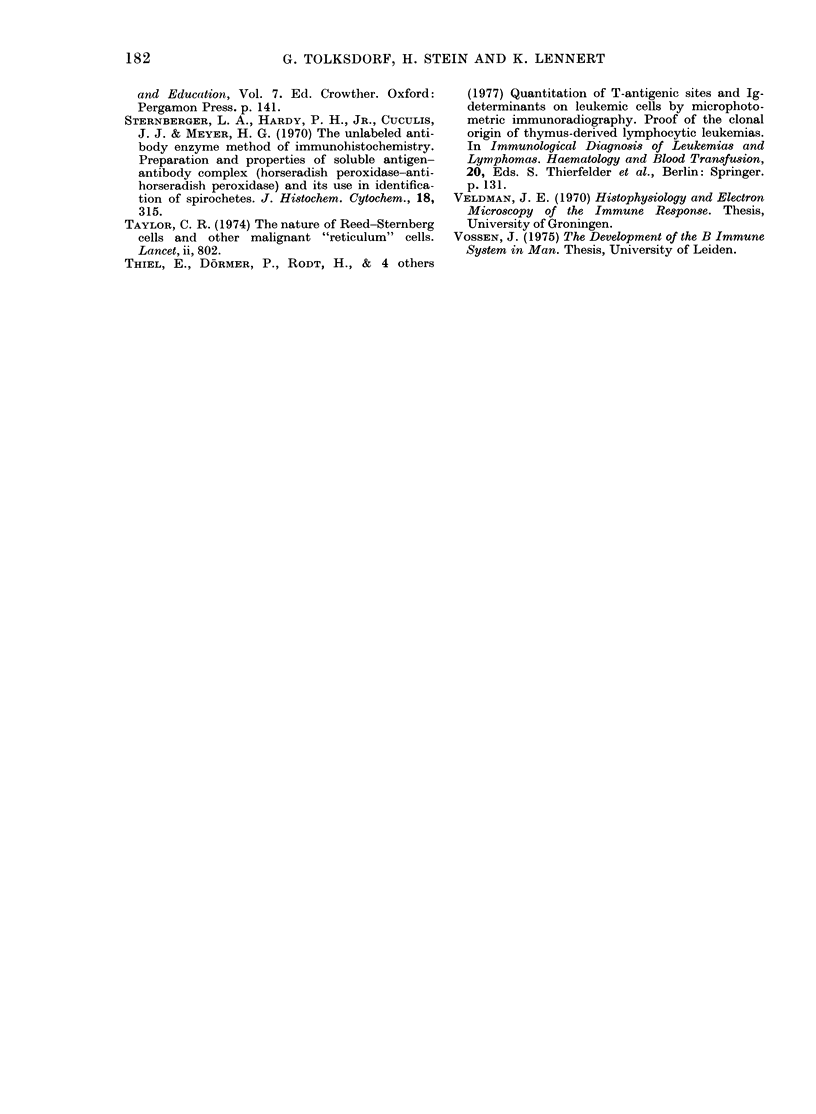# Morphological and immunological definition of a malignant lymphoma derived from germinal-centre cells with cleaved nuclei (centrocytes).

**DOI:** 10.1038/bjc.1980.27

**Published:** 1980-02

**Authors:** G. Tolksdorf, H. Stein, K. Lennert

## Abstract

**Images:**


*Br. J. Cancer* (1980) **41**, 168

### MORPHOLOGICAL AND IMMUNOLOGICAL DEFINITION OF A MALIGNANT LYMPHOMA DERIVED FROM GERMINAL-CENTRE CELLS WITH CLEAVED NUCLEI (CENTROCYTES)

G. TOLKSDORF, H. STEIN AND K. LENNERT

*From the Institute of Pathology, University of Kiel, West Germany*

Received 17 July 1979 Accepted 1 October 1979

**Summary.—Thirteen lymphomas consisting of one particular cell type were selected from 135 cases of non-Hodgkin's lymphoma. The lymphoma cells were mainly characterized by irregularly shaped nuclei and faintly stained cytoplasm. The growth pattern of the tumour was diffuse. Immunological phenotyping of suspended cells showed that the tumour cells, irrespective of whether they were isolated from lymphoma tissue or from peripheral blood of leukaemic cases, bore a dense layer of surface immunoglobulin, lacked cytoplasmic immunoglobulin and receptors for mouse erythrocytes, and expressed both complement-receptor subtypes (*i.e.*, receptors for C3b and C3d) in all but one case. The exceptional case was C3b receptor-positive and C3d receptor-negative. The number of IgG-Fc receptor-bearing cells was usually small. There was a consistently small proportion of non-malignant T cells in the tumour tissue.**

**A comparison of the properties of these lymphomas with those of other types of non-Hodgkin's lymphoma and of non-malignant lymphoid cells, shows that the cells of this type of lymphoma (a) differ morphologically and/or immunologically from the cells of all other known types of non-Hodgkin's lymphoma and (b) resemble centrocytes (cleaved follicular-centre cells) of reactive germinal centres. Thus, this type of lymphoma appears to be an entity that is closely related to, or even derived from, centrocytes.**

IN REACTIVE germinal centres, there are 2 main morphological types of lymphoid germinal-centre cells (GCC):

(1) Centroblasts—medium-sized or large cells with a round or oval, pale nucleus containing 2–3 nucleoli, usually located at the nuclear membrane; cytoplasm is sparse and intensely basophilic (Lennert, 1957, 1961).

(2) Centrocytes—small or medium-sized cells with a cleaved or irregularly shaped nucleus; cytoplasm is usually sparse and very weakly basophilic and thus difficult to recognize in sections (Lennert, 1964). Except in newly formed germinal centres, centrocytes are the predominant cells.

Both types of GCC are B cells. Most GCC express Ia-like antigen (own unpublished data). Only some GCC bear surface immunoglobulin (SIg) (Gutman & Weissman, 1972; Goldschneider & McGregor, 1973; Hoffmann-Fezer *et al.*, 1976; own unpublished data) and a few GCC contain cytoplasmic immunoglobulin (CIg); these usually resemble centrocytes (Stein & Tolksdorf in press). Characteristically, GCC express complement receptors, which was first demonstrated by Dukor *et al.* (1970). Those investigators showed that erythrocyte-antibody-complement complexes (EAC) prepared with whole mouse serum ($EAC_{mouse}$) as complement source adhered exclusively to germinal centres. Recently, our research group analysed the presence and distribution of

Address for correspondence and reprint requests: Dr H. Stein, Institute of Pathology, University of Kiel, Hospitalstr. 42, D-2300 Kiel, West Germany.

the complement-receptor subtypes (receptors for C3b and C3d) in reactive lymphoid tissue (Stein *et al.*, 1978*b*). We found that GCC bear both complement-receptor subtypes. The expression of both subtypes proved to be highly characteristic of GCC.

It has become evident that reactive germinal centres have a neoplastic equivalent, *viz.*: a lymphoma that is composed of both centroblast-like and centrocyte-like cells with a predominance of centrocyte-like cells, as have most germinal centres (Lennert, 1964, 1973; Lennert *et al.*, 1975*a*, *b*). In 95% of the cases, the tumour shows a follicular growth pattern and has thus been called "follicular lymphoma". Recently, the term "malignant lymphoma (ML) centroblastic/centrocytic" was introduced (Gérard-Marchant *et al.*, 1974; Lennert *et al.*, 1975*a*) because the cytological composition of the lymphoma is more consistent than the follicular growth pattern (which was absent in about 5% of our cases). Doubts about the close relationship between this tumour and germinal centres were eliminated by the demonstration of complement receptors on the neoplastic follicles, with a distribution and density similar to that of the complement receptors on reactive germinal centres (Jaffe *et al.*, 1974; Stein, 1976). More convincing arguments in favour of the close relationship between centroblastic/centrocytic lymphoma (CB/CC) and germinal centres were provided by the similar expression and distribution of the 2 complement-receptor subtypes in CB/CC and follicular hyperplasia (Stein *et al.*, 1978*a*, *b*).

In this paper, we describe a type of lymphoma that is probably also derived from GCC, but, in contrast to CB/CC, is composed of only one type of GCC, *i.e.*, of cells that resemble centrocytes of reactive germinal centres. We shall present immunological data that confirm the close relationship between the centrocyte-like tumour cells and reactive centrocytes. Preliminary data on this type of lymphoma have been reported in a number of previous papers (Lennert *et al.*, 1975*a*, *b*; Stein, 1976, 1978; Lennert & Mohri, 1978; Stein *et al.*, 1978*a*, 1979; Stein & Tolksdorf, 1979).

### MATERIALS AND METHODS

*Source and handling of material.*—Thirteen patients with centrocytic lymphoma, as defined by the criteria given below (see Results) were selected from a series of 135 patients with non-Hodgkin's lymphoma, from which tumour cells were studied by immunological methods. Lymphnode biopsies were available in 9 cases, together with peripheral blood in 1 of these cases, and peripheral blood was available in the other 4 cases. All 5 cases in which we investigated peripheral blood showed leukaemia (total leucocyte counts are given in Table II). After density-gradient centrifugation (see below), all lymphocyte suspensions contained a significant proportion (>85%) of tumour cells, as established by cytological and immunological analyses.

Lymphnode biopsies were also obtained from 14 patients with CB/CC, 15 with chronic lymphocytic leukaemia of B type (B-CLL), and 1 with prolymphocytic leukaemia of B type (B-PLL). Peripheral blood was available from 4 other patients with B-PLL. These disorders were diagnosed by the criteria given below (see Results).

Biopsy specimens obtained in Kiel were cut into 3 pieces immediately after surgical removal. Imprints were made from the fresh cut surface. One piece was fixed in formalin, cryosections were prepared from the second piece, and a cell suspension was prepared from the third piece. Biopsies taken in hospitals outside Kiel (up to 700 km away) were cut into 2 pieces after surgical removal and imprints were made from the fresh cut surface. One piece was placed in formalin and the other in sterile tissue-culture medium cooled with ice in a Dewar bottle. The material was then sent to our laboratory by over-night rail express. After arrival in the morning, the piece in tissue-culture medium was cut into 2 pieces when it was large enough. One of these 2 pieces was used for preparing cryosections and the other for cell suspensions.

Blood samples were supplemented with heparin (1 ml Thrombo vetren®, Promonta, Hamburg, Germany). Heparinized blood samples from outside Kiel were sent by rail express to our laboratory in 20 ml syringes cooled with ice in a Dewar bottle.

*Light microscopy.*—The piece of tissue that had been fixed in 10% formalin was embedded in paraffin. Sections were cut at 4 $\mu$m and stained with haematoxylin and eosin (H and E), Giemsa (Merck, Darmstadt, Germany) periodic-acid Schiff (PAS) and silver impregnation (Gomori). Tissue imprints were stained with Pappenheim. For cytological characterization of suspended cells and cytological identification of cells subjected to rosette assays, cytocentrifuge slides were prepared and stained with Pappenheim.

*Frozen sections.*—10 $\mu$m cryostat sections were cut from 1 part of the fresh unfixed biopsy specimen and lyophilized at $10^{-2}$ mmHg and $-68$°C. The lyophilized sections were kept at $-90$°C until use.

*Cell suspensions.*—One unfixed piece of the biopsy specimen was finely minced and passed through a plastic mesh. The filtered lymphoid cells were then separated from red blood cells, cell debris and interstitial tissue components, by density-gradient centrifugation (Bøyum, 1968). After 2 washings, the viability of the cells was measured with the trypan-blue-exclusion test. Only suspensions with 75% viable cells were used for the immunological assays. The lymphoid cell fraction was also obtained from blood samples by density-gradient centrifugation.

*Preparation of EAC intermediate complexes.*—EAC1-3b and EAC1-3d were prepared as previously described (Stein *et al.*, 1978*b*), using trypsinized sheep erythrocytes (sheep E) rabbit anti-erythrocyte antibodies of the IgM type, and functionally purified human complement components (Cordis, Miami, Florida, USA).

*Assay for binding of EAC3b and EAC3d to cells in suspensions and frozen sections.*—Target cells ($0{\cdot}75 \times 10^6$) were incubated with $3{\cdot}0 \times 10^7$ EAC in a total volume of 450 $\mu$l TC-199 medium for 10 min at 37°C and then centrifuged at 200 *g* for 30 min. The cells were then gently resuspended and kept on ice until counting. Any cell binding 3 or more indicator cells was scored as positive.

To achieve reproducible and constant binding of EAC intermediates on tissue sections, a flat chamber was built over the lyophilized sections and filled with IgM-EA, EAC-3b, or EAC-3d, as described by Stein *et al.* (1978*b*). The slides with sealed chambers were then centrifuged at 300 *g* for 8 min in a swinging rotor. The slides were washed several times in phosphate-buffered saline to remove non-adherent red blood cells. The resultant preparations were fixed for 10 min in formalin-methanol (1:9 vol/vol) stained with H. and E. and examined by transmission and dark-field light microscopy.

*Preparation of IgG-EA.*—IgG-EA was prepared by incubating ox erythrocytes (ox E) in a rabbit anti-ox-E serum, which had a low ox E agglutination titre (1:4) and a high haemolysis titre (1:2000), at 37°C for 30 min. The rabbit serum, which was rich in IgG antibodies directed against ox E, was raised by immunizing rabbits once a week i.v. with $10^{10}$ ox E for a total of 3 months. Out of 5 anti-ox-E sera, one had the desired properties.

*Assay for binding of IgG-EA.*—The same procedure as described above for binding of EAC was used.

*Mouse-erythrocyte rosette assay.*—The assay of Gupta *et al.* (1976) was applied. Briefly, $1{\cdot}5 \times 10^6$ target cells, either untreated or pretreated with neuraminidase of *Clostridium perfringens* origin (Sigma, Munich, Germany) were mixed with $10^7$ mouse erythrocytes (mouse E) and 25 $\mu$l foetal calf serum (heat-inactivated and absorbed with mouse E) in a total volume of 175 $\mu$l. The mixture was centrifuged at 200 *g* for 5 min at room temperature, followed by incubation under slight agitation at 28°C for 1 h. The pellet was gently resuspended by rotating the test tube around its long axis. Any cell that bound 3 or more mouse E was scored as positive.

*Sheep-erythrocyte rosette assay.*—The procedure described by Seiler *et al.* (1972) was followed, using sheep E treated with neuraminidase of *Vibrio cholerae* origin (Behring, Marburg, Germany).

*Ia-like antigen.*—Ia-like antigen was demonstrated by indirect fluorescence as described by Vossen (1975). Anti-Ia-like serum was obtained from Alpha Gamma Labs (Sierra Madre, California, U.S.A). The characteristics of the antiserum have been described in detail elsewhere (Billing *et al.*, 1976). Our own control experiments showed that the anti-Ia-like serum positively stained B cells, but not T cells. Goat anti-rabbit IgG conjugated with rhodamine isothiocyanate (RHITC; Nordic, Tilburg, The Netherlands) was used as the second reagent at a dilution of 1:40. When the first reagent was omitted, this goat anti-rabbit IgG-RHITC conjugate gave completely negative staining results in all tests at a dilution of 1:40.

*Surface immunoglobulin.*—SIg was detected by direct immunofluorescence, using fluorescein isothiocyanate (FITC)- and RHITC-IgG conjugates directed against human Ig chains (Nordic, Tilburg, The Netherlands). The specificity of the conjugates was tested by staining fixed cells from multiple myeloma and Waldenström's macroglobulinaemia with a known type of CIg.

*Cytoplasmic immunoglobulin.*—CIg was demonstrated on paraffin sections by means of the enzyme-bridge method of Sternberger *et al.* (1970) as modified by Taylor (1974). The procedure has been described in detail by Papadimitriou *et al.* (1978).

### RESULTS

### *Morphological findings*

*Histology.*—In the 9 cases investigated, the normal architecture of the lymph node was completely replaced by lymphoma cells. All cases showed a diffuse growth pattern. Numerous small vessels (capillaries and arterioles) were visible among the tumour cells. Epithelioid venules were generally absent. The small vessels were often surrounded by sheets of hyalin material. Thick fibres were seen, especially around the vessels.

In all cases, the vast majority of cells were small or medium-sized centrocyte-like cells (Fig. 1a). Cytoplasm was sparse and hardly visible. The cleaved or indented nuclei contained relatively sparse chromatin and often displayed one, or sometimes up to 3 small central nucleoli (Fig. 1b–d). All the tumours were completely devoid of centroblast-like cells and follicle mantle lymphocyte-like cells. In 2 tumours a small number of plasma cells of the Marschalkó type, which were obviously reactive (polyclonal Ig pattern) were present in small foci and were also scattered throughout the lymph node. In all other cases few apparently reactive plasma cells could be identified.

*Cytology.*—Cytological examination was made on tissue imprints and cytocentrifuge slides prepared from tumour-cell suspensions. The typical cytological features of the tumour cells were clearly visible in the cytocentrifuge slides, usually much better than in lymphnode imprints.

The nuclei of most tumour cells showed a characteristically irregular shape and were cleaved or sometimes even lobed (Fig. 2). They contained moderately condensed chromatin and usually up to 3 small or medium-sized pale nucleoli, which were often surrounded by a distinct rim of well-condensed chromatin. The cells had only a small amount of pale-blue cytoplasm. The tumour cells isolated from peripheral blood showed identical cytological features.

### *Immunological findings (Tables I and II)*

*Surface immunoglobulin.*—In every case studied, SIg was present on a large proportion of the tumour cells (Table I). The fluorescent staining for SIg was usually very intense. Specification of the light chains showed a restriction to one light-chain type in 6 ($\kappa$ in 5 and $\lambda$ in one case) of the 9 cases tested. In the other 3 cases, both light-chain types were detectable on tumour cells. Heavy chains ($\mu$, $\delta$, $\gamma$, and $\alpha$) were specified in 4 cases. In one case, we found none of the specified heavy chains, although most of the tumour cells showed intense staining for $\kappa$. In the other 3 cases, both $\mu$ and $\gamma$ chains were demonstrable on tumour cells. Two of the 3 $\mu$- and $\gamma$-positive cases showed staining for both $\kappa$ and $\lambda$. In the third case, the SIg was restricted to $\kappa$. Delta chains were absent from a vast majority of the tumour cells in all 4 cases.

*Cytoplasmic immunoglobulin.*—In all 9 cases investigated, the tumour cells were consistently negative for CIg (Table II). In 2 cases, there were a few $\mu$- or $\gamma$-positive plasma cells of the Marschalkó type, which were polyclonal, since they were not restricted to one light-chain type.

*Ia-like antigen.*—Ia-like antigen was present on a variable proportion (50–95%)

Fig. 1.—Histological appearance of 4 different cases of centrocytic lymphoma. (a) At intermediate magnification ( × 650) the relatively uniform cellular composition of the tumour is evident. The tumour cells have irregularly shaped or cleaved nuclei; cytoplasm is not, or only barely, visible. Centroblasts are completely absent. The arrow indicates a dendritic reticulum cell. Giemsa. (b–d) These high-power photomicrographs ( × 1050) illustrate the variation in size of the tumour cells between 3 cases, and show the nuclear features in detail. Giemsa.

FIG. 2.—Centrocytic lymphoma. The typical cytological features shown in Fig. 1 are easily recognized on cytocentrifuge slides. Tumour cells have a deeply cleaved or even lobed nucleus, sparse cytoplasm, and 1–2 small distinct nucleoli. A residual non-neoplastic lymphocyte is seen in the middle of the field. Pappenheim. ×875.

TABLE I.—*Staining for surface immunoglobulin in* 11 *cases of centrocytic lymphoma*

| Case No | Test specimen* | IgM (%) | IgD (%) | IgG (%) | IgA (%) | IgE (%) | κ (%) | λ (%) | Polyvalent (%) |
|---|---|---|---|---|---|---|---|---|---|
| 3 | LN | 83 | 0 | 80 | 0 | 0 | 50 | 46 | 95 |
| 4 | LN | 65 | 0 | 40 | 0 | 0 | 67 | 77 | 98 |
| 5 | LN | | | | | | | | 78 |
| | PB | 87 | 7 | 85 | 0 | 0 | 68 | 0 | 80 |
| 6 | LN | 0 | 3 | 0 | | | 56 | 2 | |
| 7 | LN | | | | | | | | 65 |
| 8 | LN | | | | | | 20 | 15 | |
| 9 | LN | | | | | | | | 85 |
| 10 | PB | | | | | | 100 | 31 | 100 |
| 11 | PB | | | | | | 8 | 62 | 84 |
| 12 | PB | | | | | | 94 | 3 | 70 |
| 13 | PB | | | | | | 22 | 8 | 75 |

* LN = lymph node. PB = peripheral blood.

of the tumour cells from the 4 cases tested.

*Mouse erythrocyte rosette formation.*—The proportion of mouse-E-binding tumour cells from both lymph nodes and peripheral blood was consistently small (means 0·5% and 2%, respectively). Even after pretreatment of the tumour cells with neuraminidase, the proportion of rosette-forming cells was not significantly increased (means 5·7% and 3·3%, respectively). The highest single value obtained was 17%.

*IgG-Fc receptor.*—The proportion of IgG-Fc receptor-bearing cells was generally small. Only 3 cases showed IgG-EA formation by about half of the tumour cells.

TABLE II.—*Immunological findings in 13 cases of centrocytic lymphoma*

| | | | | IgG-Fc-R | | Complement receptors: Suspension | | | Complement receptors: Frozen section | | | Mouse-E-R | | | |
|---|---|---|---|---|---|---|---|---|---|---|---|---|---|---|---|
| Case No | Test specimen | SIg (%) | CIg | EA (%) | Agg (%) | $EAC_{mo}$ (%) | EAC3b (%) | EAC3d (%) | $EAC_{mo}$ | EAC3b | EAC3d | Ly (%) | LyN (%) | Ia (%) | Sheep-E-R (%) |
| 1 | LN | | – | | | | | | + | + | + | | | | |
| 2 | LN | | – | | | | | | | + | – | | | | |
| 3 | LN | 95 | – | | 20 | 67 | 40 | 29 | + | + | + | 0 | 1 | | 9 |
| 4 | LN | 98 | – | 0 | | | 84 | 62 | + | + | + | | 17 | | 14 |
| 5 | LN | 78 | – | 0 | | | 36 | 7 | | | | 1 | | | 14 |
| 6 | LN | 56 | – | 0 | | 81 | 72 | 76 | | | | 2 | 16 | | 7 |
| 7 | LN | 65 | – | 12 | | | 74 | 72 | | | | 0 | 0 | | 7 |
| 8 | LN | | – | 0 | | | 56 | 67 | | | | 0 | 0 | | 3 |
| 9 | LN | 85 | – | 40 | | | 76 | 80 | | | | 0 | 0 | 90 | 2 |
| Mean | | 79·5 | | 8·7 | 20 | 74 | 62·5 | 56·1 | | | | 0·5 | 5·7 | 90 | 8·0 |
| s.d. | | 16·6 | | 16·1 | | | 18·8 | 27·4 | | | | 0·8 | 8·4 | | 4·8 |
| 5 | PB (35,700)* | 87 | | 17 | 10 | 28 | 18 | 17 | | | | 0 | 0 | | 0 |
| 10 | PB (23,000) | 95 | | 10 | | | 75 | 55 | | | | 0 | | | 3 |
| 11 | PB (29,000) | 84 | | 56 | | | 47 | 68 | | | | 0 | 0 | 50 | 13 |
| 12 | PB (21,000) | 94 | | 46 | | | 77 | 93 | | | | 8 | 8 | 73 | 5 |
| 13 | PB (18,000) | 75 | | 39 | | | 28 | 14 | | | | 0 | 2 | 72 | 4 |
| Mean | | 87·0 | | 33·6 | 10 | 28 | 49·0 | 49·4 | | | | 1·6 | 2·5 | 65·0 | 5·0 |
| s.d. | | 8·2 | | 19·5 | | | 26·8 | 33·8 | | | | 3·6 | 3·8 | 13·0 | 4·8 |
| Total mean† | | 82·5 | | 20·3 | 20 | 74 | 60·5 | 56·6 | | | | 1·1 | 4·9 | 71·3 | 7·4 |
| s.d. | | 14·1 | | 22·4 | | | 19·7 | 27·9 | | | | 2·5 | 7·1 | 16·4 | 4·5 |

* Leucocytes/$mm^3$.

† Excluding Case 5 (PB).

Abbreviations: SIg = surface immunoglobulin. CIg = cytoplasmic immunoglobulin. IgG-Fc-R = IgG-Fc receptor. EA = ox erythrocytes densely coated with rabbit IgG. Agg = aggregated human IgG. $EAC_{mo}$ = trypsinized sheep erythrocytes coated with rabbit IgM and mouse complement. EAC3b = trypsinized sheep erythrocytes coated with rabbit IgM and purified human C3b. EAC3d = C3b inactivator-cleaved EAC3b. Mouse-E-R = mouse erythrocyte receptor. Ly = untreated lymphocytes. LyN = lymphocytes pretreated with neuraminidase. Ia = Ia-like antigen. Sheep-E-R = sheep erythrocyte receptor. LN = lymph node. PB = peripheral blood.

*Complement receptors and complement-receptor subtypes.*—Complement receptors were demonstrable by EAC-rosette assay in all 11 cases tested. Specification of the complement-receptor subtypes (C3b and C3d) revealed that both subtypes were present on most of the suspended cells, from all but one case. In the exceptional case, EAC3b-binding cells predominated in the lymphnode cell suspension, whereas about equal proportions of EAC3b- and EAC3d-binding cells were found among the circulating neoplastic cells (Table II, case 5). Altogether, the mean percentage of EAC3b-binding cells (62%) was slightly higher than that of EAC3d rosette-forming cells (56%) in lymphnode suspensions. In the 4 cases showing a leukaemic blood picture, analysis of the leukaemic blood cells revealed 56% EAC3b- and 57% EAC3d-binding cells. Cytological evaluation of the rosette-forming cells of cytocentrifuge slides demonstrated the typical morphological features of small or medium-sized cleaved cells described above.

The adherence of EAC3b and EAC3d to frozen sections was investigated in 4 cases. Both EAC3b and EAC3d adhered to the sections in 3 of them. In the fourth case, only adherence of EAC3b was demonstrable. The adherent EAC were distributed in a nodular pattern in all 4 cases.

*Sheep erythrocyte rosette formation.*—The percentage of cells showing spontaneous sheep E-rosette formation was consistently low (means 7% of suspended lymphnode cells and 8% of the mononuclear-cell fraction from leukaemic cases) never exceeding 14%. On cytological examination, the sheep E-binding cells were identifiable as small lymphocytes. Tumour cells failed to bind sheep E.

*Differential diagnosis*

We compared the findings in centrocytic lymphoma with the results of morphological and immunological investigations on a total of 34 cases of 3 types of non-Hodgkin's lymphoma that may be confused with centrocytic lymphoma: CB/CC (14), B-CLL (15), and B-PLL (5).

*Histology.*—CB/CC, which is roughly equivalent to follicular lymphoma, imitates follicular hyperplasia. Centroblasts and centrocytes always proliferate side by side, but with a predominance of centrocytes. In B-CLL, the proliferating cells are lymphocytes and a few larger cells, which are usually grouped together to form light-appearing areas that are highly characteristic of B-CLL. Such light areas are not seen in T-CLL. We diagnose B-PLL by the criteria given by Galton *et al.* (1974). Two out of the 5 cases of B-PLL revealed tartrate-resistant acid-phosphatase activity in a variable proportion of the leukaemic cells.

*Immunological findings.*—The immunological data on CB/CC, B-CLL, B-PLL, and centrocytic lymphoma are summarized in Table III.

*Surface immunoglobulin.*—Thirteen cases of CB/CC were investigated for the presence of SIg. All cases were positive, with a mean of 67% reactive cells among the suspended lymphoma cells. The fluorescence was usually easily detectable. Twelve cases of B-CLL were analysed and all showed positive SIg staining, with a mean of 60% of the suspended cells. The fluorescence was generally weak and often barely detectable. In all 5 cases of B-PLL, a large proportion of the suspended cells (mean 86%) showed bright fluorescence when stained with anti-Ig sera.

*Cytoplasmic immunoglobulin.*—Thirteen cases of CB/CC were tested for CIg. In all, at least a few plasma cells with positive staining for both light chain types ($\kappa$ and $\lambda$) were found in parafollicular regions. The tumour cells in the neoplastic follicles were completely negative in all but 2 cases, in which a few intrafollicular cells, whose nuclei were identical to those of the $CIg^-$ intrafollicular cells, showed positive staining that was definitely restricted to one light-chain type. In all 15 cases of B-CLL, the tumour cells were $CIg^-$. Two cases of B-PLL were tested for CIg and both were also negative.

*Ia-like antigen.*—In the 3 CB/CC cases tested, a mean of 62% of the suspended tumour cells reacted with antiserum specific for HL-B antigen (Ia-like antigen). The 3 tested cases of B-CLL showed a mean of 48% positively stained tumour cells. Two cases of B-PLL revealed a mean percentage of 60% positively reacting cells.

*Mouse erythrocyte rosette formation.*—In 14 cases of CB/CC, the mean proportion of mouse E rosette-forming cells was 15% of the suspended tumour cells. Pretreatment of the lymphocytes with neuraminidase in 13 cases increased the mean to 29%. The mean percentage of mouse E rosette-forming cells in 15 cases of B-CLL was 44% without, and 62% with pretreatment of the lymphocytes with neuraminidase. Five cases of B-PLL were tested without and with pretreatment of the lymphocytes with neuraminidase. The mean percentage of mouse E rosette-forming cells was 2% and 7%, respectively.

*IgG-Fc receptor.*—In the 12 cases of CB/CC tested, the proportion of cells forming rosettes with IgG-coated ox E was generally small (mean: 15%). Eight cases were tested with FITC-conjugated IgG aggregates and a mean of 10% of the cells showed positive fluorescence. Fourteen cases of B-CLL were investigated with ox E coated with IgG. The mean percentage of rosette-forming cells was 19%. With aggregated IgG, the mean percentage of positively reacting cells was higher (mean of 47% for 7 cases). The average frequency of IgG-EA rosette-forming cells in 5 cases of B-PLL was 36%.

*Complement receptors and complement-receptor subtypes.*—Complement receptors were demonstrable in all cases of CB/CC tested. In 9 cases, a mean of 48% of the suspended cells showed rosette formation with $EAC_{mouse}$. Complement receptor subtypes were analysed in all 14 cases. The mean percentage of EAC3b-binding cells was 33% and of EAC3d-binding cells 38%. The $EAC_{mouse}$ rosette assay in 7 cases of B-CLL revealed a mean of 69·9% rosette-forming cells. Complement-receptor subtypes were specified in all 15 cases. There was a predominance of C3d receptor-bearing cells (mean 66%) over C3b receptor-bearing cells (mean 22·9%). Complement receptors were detectable in all tested cases of B-PLL. There was considerable variation, however, in the number of EAC-binding cells when the complement-receptor subtypes were analysed. Nevertheless, the average number of $C3b^+$ cells exceeded the number of cells with C3d receptors.

*Sheep erythrocyte rosette formation.*—The proportion of sheep E-rosette-forming cells was usually large (mean 22%) in the 14 cases of CB/CC. The rosette-forming cells were cytologically identifiable as small lymphocytes. In the 15 cases of B-CLL, a mean of 8% of the suspended cells formed rosettes with sheep E. In B-PLL, the proportion of sheep E-rosette-forming cells depended on the number of prolymphocytes in the blood, and was thus usually small (mean 10%). The lymphnode cell suspension from one case of B-PLL revealed 18% rosette-forming cells.

### DISCUSSION

The main morphological criterion of the lymphoma discussed here is the nuclear polymorphism of the tumour cells. The nuclei are irregularly shaped, with deep clefts or anguli, which are most easily recognizable on histological sections and on cytocentrifuge slides prepared from tumour-cell suspensions. Electron-microscopical studies have confirmed that the irregular nuclear shape is not an artifact (Kaiserling, 1978). The nuclear chromatin is less condensed than that of lymphocytes. Up to 3 small central nucleoli are visible. With Giemsa staining, the cytoplasm is often too faint to be visible. The tumour cells range in size from small to intermediate. Comparing the cytological features of these tumour cells with those of the various cells present in non-malignant lymphoid tissue, it is obvious that the tumour cells are most similar in morphology to centrocytes of reactive germinal centres. The tumour shows a diffuse growth pattern in all but rare cases. In those exceptional cases, a vaguely nodular pattern is recognizable, especially with Gomori staining. All the cases in the present investigation showed a diffuse growth pattern.

The lymphomas with the above morphological characteristics revealed remarkably uniform immunological features, whether tumour cells from lymph nodes or from peripheral blood were examined. After labelling of the cells with fluorescent anti-Ig sera, the usually strong fluorescence reflected relatively large amounts of SIg. In 6/9 cases, the staining pattern was clearly monotypic (positive for only one light-chain type), in agreement with previously reported data on 14 cases (Stein, 1978). In that study, the SIg staining was restricted to one light-chain type in only 9 of the 14 cases. Other authors have reported large amounts of SIg restricted to one light-chain type in all or most cases of ML poorly differentiated (Hopper, 1974; Preud'Homme *et al.*, 1974; Aisenberg & Long, 1975; Brouet *et al.*, 1975) ML intermediate differentiation (Berard *et al.*, 1978) or follicular-centre cell (FCC) lymphoma, small and large cleaved, diffuse (Leech *et al.*, 1975). At least some of the cases described by those authors are equivalent to the type of lymphoma under discussion here. Like the tumour cells of CB/CC (follicular lymphoma), those of the lymphoma type described here bore a relatively dense layer of SIg. Reactive GCC usually do not show such a high density of SIg. The reason for this discrepancy has not yet been found.

In all cases stained for CIg by the PAP method, the tumour cells were consistently negative, indicating no synthesis of secretory Ig. This finding concurs with the lack of secretory organelles (ergastoplasm) in the tumour cells (Kaiserling, 1978) and the usually decreased levels of serum Ig (Stein, 1976).

In the 4 cases tested, Ia-like antigen was demonstrable in a variable proportion of the cells. The presence of Ia-like antigen and SIg proves the B-cell nature of the tumour cells.

The proportion of mouse E-rosette-forming cells was consistently small, even after treatment of the tumour cells with neuraminidase. This finding agrees with data on cases of lymphosarcoma-cell leukaemia reported by Catovsky *et al.* (1976); lymphosarcoma-cell leukaemia might be roughly equivalent to the type of lymphnode tumour described in this report. In their inability to bind mouse E, the tumour cells resemble centrocytes of reactive germinal centres (own unpublished data).

Complement receptors were expressed in all 13 cases analysed. Complement-receptor subtypes (receptors for C3b and C3d) were specified in 11 of those, in all of which both complement-receptor subtypes could be demonstrated on the tumour cells. The percentages of C3b receptor-positive and C3d receptor-positive tumour cells were about equal in all but one case. In that exception, there was a definite preponderance of EAC3b-reactive cells over EAC3d-reactive cells. These findings, obtained on cell suspensions, agree with our earlier data (Stein & Tolksdorf, 1979) and with the demonstration of complement-receptor subtypes on frozen tissue sections from 4 cases. In 3 of those both EAC3b and EAC3d, and in the fourth, only EAC3b adhered to the sections. We may conclude from the results presented here that (a) the simultaneous presence of both complement-receptor subtypes is highly characteristic of the lymphoma cells under discussion, and (b) occasionally, C3d receptors are decreased in number, or even absent.

The presented data on complement-receptor subtypes are interesting in connection with our recent observations on the occurrence and distribution of complement receptor subtypes in follicular hyperplasia (Stein *et al.*, 1978*b*). We found that EAC3d usually adhered to germinal centres and the follicular mantle, whereas EAC3b adhered not only to germinal centres and the follicular mantle, but also to parafollicular areas and, in many instances, to interfollicular areas, but spared the T-dependent paracortical zone. In rare instances, however, we observed germinal centres with small areas to which only EAC3b and no, or reduced amounts of, EAC3d were bound. Cytological evaluation of EAC3b- and EAC3d-binding tonsil cells on cytocentrifuge slides confirmed that cells exhibiting the typical features of GCC usually express both complement-receptor subtypes. Taking the data on follicular hyperplasia into consideration, we may conclude that the lymphomas showing both complement receptor subtypes are most likely derived from GCC. The cells of the 2 cases that showed a predominance or exclusive expression of C3b receptors might be related to GCC that express only C3b and no, or only reduced amounts of, C3d receptors.

Further evidence of a relationship between the type of lymphoma cell described here and GCC has been provided by electron-microscopical investigations (Kaiserling, 1978). In 12/20 isomorphic cases, dendritic reticulum cells were demonstrable among the tumour cells. Dendritic reticulum cells appear to be restricted to the lymphnode cortex, in which the germinal-centre reaction takes place (Milanesi, 1965; Mitchell & Abbot, 1965; Nossal *et al.*, 1968; Lennert & Niedorf, 1969; Veldman, 1970).

In conclusion, both the morphological and the immunological findings suggest that the type of lymphoma described here is a separate entity. It appears to be a monoclonal proliferation of the GCC that we call "centrocytes", which led to the term "ML centrocytic".

Centrocytic lymphoma, as it is described here, is not found as a separate entity in other classifications. In former German and other European classification concepts, it was often grouped together with other lymphomas under the term "lymphocytic lymphosarcoma" and the leukaemic cases were placed under "leukosarcomatosis" or sometimes "CLL". According to Rappaport's (1966) classification, cases of centrocytic lymphoma would be diagnosed as either ML lymphocytic, well differentiated, or ML lymphocytic, poorly differentiated. In the classification of Lukes & Collins (1975), centrocytic lymphoma is included among the FCC lymphomas, small and large cleaved types. Thus, Lukes & Collins' interpretation comes close to our own. In contrast to our concept, however, they define the small and large cleaved types of FCC lymphoma as those malignant lymphomas in which the number of cleaved cells (centrocytes) exceeds the number of non-cleaved cells (centroblasts) by a factor of 3. The small and large cleaved types of FCC lymphoma of Lukes & Collins are thus composed either of a mixture of centroblasts and centrocytes with a predominance of centrocytes, or of centrocytes alone. In other words, FCC lymphomas of the cleaved type include both CB/CC and centrocytic lymphoma of our classification concept. That explains why most small and large cleaved FCC lymphomas of Lukes & Collins exhibit a follicular growth pattern. In contrast, centrocytic lymphoma usually has a diffuse growth pattern.

Berard *et al.* (1978) have distinguished a type of malignant lymphoma, which they called "ML intermediate differentiation" (MLID), that shares some morphological and immunological features with centrocytic lymphoma. The authors described the growth pattern of MLID as usually diffuse, but sometimes vaguely nodular. The tumour cells were small and relatively uniform in appearance. They showed a high density of SIg, and most cells had complement receptors, as do centrocytic lymphoma cells. In contrast to our cases, however, in which nearly all tumour cells had more or less cleaved nuclei, the nuclei in MLID were described as a variable mixture of small round ones (resembling those of well-differentiated lymphocytic lymphoma or B-CLL) and cleaved ones (like those of follicular lymphoma). Thus, the term "ML intermediate differentiation" is applied to a type of lymphoma that consists of 2 different types of cells. Three out of the 6 cases of MLID studied by Berard and co-workers were alkaline-phosphatase-positive. We did not perform the alkaline phosphatase reaction in our series of cases. It will be necessary to perform further studies to clarify the relationship between MLID and centrocytic lymphoma.

A comparison of the properties of centrocytic lymphoma with those of CB/CC, or follicular lymphoma, which is now generally thought to be a GCC-derived lymphoma (Jaffe *et al.*, 1974; Lukes & Collins, 1975; Stein *et al.*, 1978*b*) justifies separating these types of lymphoma. Briefly, CB/CC consistently differs from centrocytic lymphoma in the composition of the tumour; CB/CC is a mixed proliferation of centrocytes (predominant in number) and centroblasts. CB/CC also differs from centrocytic lymphoma in the follicular growth pattern, the presence of a number of mouse E-rosetting cells, and the presence of a relatively large proportion of T cells (see Table III). There is also a difference in prognosis: patients with CB/CC survive longer than patients with centrocytic lymphoma (Brittinger, 1978).

Centrocytic lymphoma is also clearly distinguishable from B-CLL. In B-CLL, the predominant type of cell resembles blood lymphocytes, which usually have a round or oval, and relatively regularly shaped nucleus. Among the lymphocytes, there are always some medium-sized cells and a few large blast cells with a central nucleolus; the medium-sized and large cells are often grouped in clusters and thus produce a so-called pseudofollicular pattern. Immunologically, B-CLL cells differ from the cells of centrocytic lymphoma in the usually low density of SIg, the large proportion of mouse E-

TABLE III. *Immunological findings in centrocytic lymphoma, centroblastic/centrocytic lymphoma, chronic lymphocytic leukaemia of B type, and prolymphocytic leukaemia of B type*

| | | | IgG-Fc-R | | Complement receptors | | | Mouse-E-R | | | |
|---|---|---|---|---|---|---|---|---|---|---|---|
| | SIg | CIg* | EA | Agg | $EAC_{mo}$ | EAC3b | EAC3d | Ly | LyN | Ia | Sheep-E-R |
| Centrocytic lymphoma | | | | | | | | | | | |
| No. of cases | 10 | 9 | 10 | 1 | 2 | 11 | 11 | 10 | 9 | 4 | 11 |
| Mean (%)* | 82·5 | 0 | 20·3 | 20 | 74 | 60·5 | 56·6 | 1·1 | 4·9 | 71·3 | 7·4 |
| s.d. | 14·1 | | 22·4 | | | 19·7 | 27·9 | 2·5 | 7·1 | 16·4 | 4·5 |
| Centroblastic/centrocytic lymphoma | | | | | | | | | | | |
| No. of cases | 13 | 13 | 12 | 8 | 9 | 14 | 14 | 14 | 13 | 3 | 14 |
| Mean (%) | 67·3 | 3 | 15 | 10·8 | 48·6 | 33·4 | 38·1 | 15·7 | 29·6 | 62·7 | 22 |
| s.d. | 20·1 | | 18·4 | 12 | 15·9 | 13·7 | 16·3 | 17·2 | 19 | 11·7 | 7·7 |
| Chronic lymphocytic leukaemia | | | | | | | | | | | |
| No. of cases | 12 | 15 | 14 | 7 | 7 | 15 | 15 | 15 | 14 | 3 | 15 |
| Mean (%) | 60·6 | 0 | 19·2 | 47 | 69·9 | 22·9 | 66·2 | 44·1 | 62·8 | 48·7 | 8·3 |
| s.d. | 24·6 | | 19·6 | 23·7 | 13·2 | 15·3 | 15 | 21·8 | 17·2 | 15·5 | 2·8 |
| Prolymphocytic leukaemia | | | | | | | | | | | |
| No. of cases | 5 | 2 | 5 | 0 | 1 | 5 | 5 | 5 | 5 | 2 | 5 |
| Mean (%) | 86·8 | 0 | 36·8 | | 80 | 40·6 | 29·4 | 2·6 | 7·2 | 60 | 10·2 |
| s.d. | 16·5 | | 33·6 | | | 29·1 | 27·0 | 4·7 | 10·1 | | 6·1 |

* No. of cases containing $CIg^+$ tumour cells is shown instead of mean %.
Abbreviations as in Table II.

rosetting cells, and the predominance of C3d receptor-bearing cells over C3b receptor-bearing cells (see Table III). Furthermore, B-CLL and centrocytic lymphoma differ in clinical behaviour and prognosis (Brittinger, 1978).

B-PLL is the only disorder that is not always clearly distinguishable from centrocytic lymphoma by immunological methods. The tumour cells of B-PLL bear a dense layer of SIg, lack CIg, express a variable number of complement receptors of both subtypes and lack mouse E-receptors; B-PLL thus resembles centrocytic lymphoma immunologically (see Table III). B-PLL has to be differentiated from centrocytic lymphoma by cytological criteria and clinical behaviour, or by detection of tartrate-resistant acid phosphatase activity.

Centrocytic lymphoma is easily distinguishable morphologically from all other types of non-Hodgkin's lymphoma of B-cell type that were not discussed here, *viz.*: lymphoplasmacytic/-cytoid lymphoma (LP immunocytoma), lymphoblastic lymphoma of the Burkitt type and of the unclassified type, and immunoblastic lymphoma (large-cell lymphoma). It should be mentioned, however, that the morphological differential diagnosis between centrocytic lymphoma and lymphoblastic lymphoma of the convoluted-cell type is sometimes difficult, because the "convolutions" of the nuclei in lymphoblastic lymphoma of the convoluted-cell type are not always clearly distinguishable in histological sections from the "cleavage" or "indentations" of the nuclei in centrocytic lymphoma. In such cases, these 2 types of lymphoma can be clearly differentiated by the acid-phosphatase reaction, or the more reliable immunological assays: lymphoblastic lymphoma of the convoluted-cell type shows focal acid-phosphatase activity and is $SIg^-$ and sheep E-rosette-positive in most cases, and is human T-lymphocyte antigen-positive in all cases (Stein *et al.*, 1976, 1979; Thiel *et al.*, 1977).

In summary, the morphological and immunological data presented here and compared with those on other known entities, not only justify, but compel the separation of the type of lymphoma described in this paper as a distinct entity. Since the tumour cells are highly similar to centrocytes of reactive germinal centres (cleaved nuclei, presence of SIg and both complement-receptor subtypes, and absence of receptors for mouse E) we consider this lymphoma entity to be closely related to, or even derived from, centrocytes.

This work was supported by the Deutsche Forschungsgemeinschaft, SFB 111, Project CL1.

The authors wish to thank Mrs M. Soehring for preparing the manuscript.

### REFERENCES

Aisenberg, A. C. & Long, J. C. (1975) Lymphocyte surface characteristics in malignant lymphoma. *Am. J. Med.*, **58**, 300.

Berard, C. W., Jaffe, E. S., Braylan, R. C., Mann, R. B. & Nanba, K. (1978) Immunologic aspects and pathology of the malignant lymphomas. *Cancer*, **42**, 911.

Billing, R., Rafizadeh, B., Drew, I., Hartman, G., Gale, R. & Terasaki, P. (1976) Human B-lymphocyte antigens expressed by lymphocytic and myelocytic leukemia cells. I. Detection by rabbit antisera. *J. Exp. Med.*, **144**, 167.

Bøyum, A. (1968) Isolation of mononuclear cells and granulocytes from human blood. *Scand. J. Clin. Lab. Invest.*, *Suppl.* **97**, 77.

Brittinger, G. (1978) Outline of a prospective multicentric study on the clinical significance of the Kiel classification of non-Hodgkin's lymphomas. *Recent Results Cancer Res.*, **65**, 197.

Brouet, J. C., Labaume, S. & Seligmann, M. (1975) Evaluation of T and B lymphocyte membrane markers in human non-Hodgkin malignant lymphomata. *Br. J. Cancer*, **31**, *Suppl.* II, 121.

Catovsky, D., Cherchi, M., Okos, A., Hedge, U. & Galton, D. A. G. (1976) Mouse red-cell rosettes in B-lymphoproliferative disorders. *Br. J. Haematol.*, **33**, 173.

Dukor, P., Bianco, C. & Nussenzweig, V. (1970) Tissue localization of lymphocytes bearing a membrane receptor for antigen-antibody-complement complexes. *Proc. Natl Acad. Sci. U.S.A.*, **67**, 991.

Galton, D. A. G., Goldman, J. M., Wiltshaw, E., Catovsky, E., Henry, K. & Goldenberg, G. J. (1974) Prolymphocytic leukaemia. *Br. J. Haematol.*, **27**, 7.

Gérard-Marchant, R., Hamlin, I., Lennert, K., Rilke, F., Stansfeld, A. G. & van Unnik, J. A. M. (1974) Classification of non-Hodgkin's lymphomas. *Lancet*, ii, 406.

Goldschneider, I. & McGregor, D. D. (1973) Anatomical distribution of T and B lymphocytes in the rat. Development of lymphocyte-specific antisera. *J. Exp. Med.*, **138**, 1443.

GUPTA, S., GOOD, R. A. & SIEGAL, F. P. (1976) Rosette formation with mouse erythrocytes. II. A marker for human B and non-T lymphocytes. *Clin. Exp. Immunol.*, **25**, 319.

GUTMAN, G. A. & WEISSMAN, I. L. (1972) Lymphoid tissue architecture: Experimental analysis of the origin and distribution of T-cells and B-cells. *Immunology*, **23**, 465.

HOFFMANN-FEZER, G., RODT, H., EULITZ, M. & THIERFELDER, S. (1976) Immunohistochemical identification of T- and B-lymphocytes delineated by the unlabeled antibody enzyme method. I. Anatomical distribution of $\theta$-positive and Ig-positive cells in lymphoid organs of mice. *J. Immunol. Methods*, **13**, 261.

HOPPER, J. E. (1974) Immunoglobulin D: A predominant surface Ig in poorly differentiated lymphocytic (PDL) lymphoma. *Clin. Res.*, **22**, 394A.

JAFFE, E. S., SHEVACH, E. M., FRANK, M. M., BERARD, N. C. & GREEN, I. (1974) Nodular lymphoma—evidence for origin from follicular B lymphocytes. *N. Engl. J. Med.*, **290**, 813.

KAISERLING, E. (1978) Ultrastructure of non-Hodgkin's lymphomas. In *Malignant Lymphomas Other than Hodgkin's Disease*, Eds. Lennert *et al.* New York: Springer. p. 471.

LEECH, J. H., GLICK, A. D., WALDRON, J. A., FLEXNER, J. M., HORN, R. G. & COLLINS, R. D. (1975) Malignant lymphomas of follicular center cell origin in man. I. Immunologic studies. *J. Natl Cancer Inst.*, **54**, 11.

LENNERT, K. (1957) Über die Erkennung von Keimzentrumszellen im Lymphknotenausstrich. *Klin. Wochenschr.*, **35**, 1130.

LENNERT, K. (1961) Lymphknoten. Diagnostik in Schnitt und Ausstrich. Bandteil A: Cytologie und Lymphadenitis. In *Handbuch der speziellen pathologischen Anatomie und Histologie*, Vol. I, Part 3A. Eds. O. Lubarsch *et al.* Berlin: Springer. p. 66.

LENNERT, K. (1964) *Pathologie der Halslymphknoten. Ein Abriß für Pathologen, Kliniker und praktizierende Ärzte.* Berlin: Springer. p. 68.

LENNERT, K. (1973) Follicular lymphoma. A tumor of the germinal centers. In *Malignant Diseases of the Hematopoietic System. GANN Monograph on Cancer Research*, Vol. **15**, Eds. K. Akazaki *et al.* Tokyo: University of Tokyo Press. p. 217.

LENNERT, K. & MOHRI, N. (1978) Histopathology and diagnosis of non-Hodgkin's lymphomas. In *Malignant Lymphomas Other than Hodgkin's Disease*. Eds. Lennert *et al.* New York: Springer. p. 111.

LENNERT, K., MOHRI, N., STEIN, H. & KAISERLING, E. (1975*a*) The histopathology of malignant lymphoma. *Br. J. Haematol.*, **31** (Suppl.), 193.

LENNERT, K. & NIEDORF, H. R. (1969) Nachweis von desmosomal verknüpften Reticulumzellen im follikulären Lymphom (Brill-Symmers). *Virchows Archiv [Cell Pathol.]*, **4**, 148.

LENNERT, K., STEIN, H. & KAISERLING, E. (1975*b*) Cytological and functional criteria for the classification of malignant lymphomata. *Br. J. Cancer*, **31**, Suppl. II, 29.

LUKES, R. J. & COLLINS, R. D. (1975) New approaches to the classification of the lymphomata. *Br. J. Cancer*, **31**, Suppl. II, 1.

MILANESI, S. (1965) Sulla presenza di dispositivi di giunzione tra le cellule dendritiche dei follicoli linfatici del linfonodo. *Boll. Soc. Ital. Biol. Sper.*, **41**, 1223.

MITCHELL, J. & ABBOT, A. (1965) Ultrastructure of the antigen-retaining reticulum of lymph node follicles as shown by high-resolution autoradiography. *Nature*, **208**, 500.

NOSSAL, G. J. V., ABBOT, A., MITCHELL, J. & LUMMUS, Z. (1968) Antigens in immunity. XV. Ultrastructural features of antigen capture in primary and secondary lymphoid follicles. *J. Exp. Med.*, **127**, 277.

PAPADIMITRIOU, C. S., STEIN, H. & LENNERT, K. (1978) The complexity of immunohistochemical staining pattern of Hodgkin and Sternberg–Reed cells—Demonstration of immunoglobulin, albumin, $\alpha_1$-antichymotrypsin and lysozyme. *Int J. Cancer*, **21**, 531.

PREUD'HOMME, J. L., BROUET, J. C., CLAUVEL, J. P. & SELIGMANN, M. (1974) Surface IgD in immunoproliferative disorders. *Scand. J. Immunol.* **3**, 853.

RAPPAPORT, H. (1966) *Tumors of the Hematopoietic System. Atlas of Tumor Pathology, Sect., Fasc.* 8. Washington, D.C.: Armed Forces Institute of Pathology.

SEILER, F. R., SEDLACEK, H. H., KANZY, E. J. & LANG, W. (1972) Über die Brauchbarkeit immunologischer Nachweismethoden zur Differenzierung funktionell verschiedener Lymphozyten: Spontanrosetten, Komplementreceptor-Rosetten und Immunglobulin-rezeptoren. *Behring Inst. Mitt.*, **52**, 26.

STEIN, H. (1976) Klassifikation der malignen Non-Hodgkin-Lymphome aufgrund gemeinsamer morphologischer und immunologischer Merkmale zwischen normalen und neoplastischen lymphatischen Zellen. *Immun. Infekt.*, **4**, 52.

STEIN, H. (1978) The immunologic and immunochemical basis for the Kiel classification. In *Malignant Lymphomas other than Hodgkin's Disease*, Eds. Lennert *et al.* New York: Springer. p. 529.

STEIN, H., PAPADIMITRIOU, C. S., BOUMAN, H., LENNERT, K. & FUCHS, J. (1978*a*) Demonstration of immunoglobulin production by tumor cells in non-Hodgkin's and Hodgkin's malignant lymphomas and its significance for their classification. *Recent Results Cancer Res.*, **64**, 158.

STEIN, H., PETERSEN, N., GAEDICKE, G., LENNERT, K. & LANDBEK, G. (1976) Lymphoblastic lymphoma of convoluted or acid phosphatase type—a tumor of T precursor cells. *Int. J. Cancer*, **17**, 292.

STEIN, H., SIEMSSEN, U. & LENNERT, K. (1978*b*) Complement receptor subtypes C3b and C3d in lymphatic tissue and follicular lymphoma. *Br. J. Cancer*, **37**, 520.

STEIN, H. & TOLKSDORF, G. (1979) Die immunologische Basis der Kiel-Klassifikation der malignen Non-Hodgkin-Lymphome. In *Lymphknotentumoren*, Ed. A. Stacher & P. Höcker. München: Urban & Schwarzenberg. p. 108.

STEIN, H., TOLKSDORF, G., BURKERT, M. & LENNERT, K. (1979) Cytologic classification of non-Hodgkin's lymphomas based on morphology, cytochemistry, and immunology. In *Leukemia and Non-Hodgkin Lymphoma. Advances in Medical Oncology, Research*

*and Education*, Vol. 7. Ed. Crowther. Oxford: Pergamon Press. p. 141.

STERNBERGER, L. A., HARDY, P. H., JR., CUCULIS, J. J. & MEYER, H. G. (1970) The unlabeled antibody enzyme method of immunohistochemistry. Preparation and properties of soluble antigen–antibody complex (horseradish peroxidase–antihorseradish peroxidase) and its use in identification of spirochetes. *J. Histochem. Cytochem.*, **18**, 315.

TAYLOR, C. R. (1974) The nature of Reed–Sternberg cells and other malignant "reticulum" cells. *Lancet*, ii, 802.

THIEL, E., DÖRMER, P., RODT, H., & 4 others (1977) Quantitation of T-antigenic sites and Ig-determinants on leukemic cells by microphotometric immunoradiography. Proof of the clonal origin of thymus-derived lymphocytic leukemias. In *Immunological Diagnosis of Leukemias and Lymphomas. Haematology and Blood Transfusion*, **20**, Eds. S. Thierfelder *et al.*, Berlin: Springer. p. 131.

VELDMAN, J. E. (1970) *Histophysiology and Electron Microscopy of the Immune Response*. Thesis, University of Groningen.

VOSSEN, J. (1975) *The Development of the B Immune System in Man*. Thesis, University of Leiden.